# How do middle-aged patients and their healthcare providers manage multimorbidity? Results of a qualitative study

**DOI:** 10.1371/journal.pone.0291065

**Published:** 2023-08-31

**Authors:** Truc Sophia Dinh, Robin Brünn, Christine Schwarz, Maria-Sophie Brueckle, Mirjam Dieckelmann, Ana I. González González, Marjan van den Akker

**Affiliations:** 1 Institute of General Practice, Goethe University, Frankfurt am Main, Germany; 2 Instituto de Salud Carlos III, Madrid, Spain; 3 Red de Investigación en Cronicidad, Atención Primaria y Promoción de la Salud (RICAPPS), Madrid, Spain; 4 Department of Family Medicine, School CAPHRI, Maastricht University, Maastricht, The Netherlands; 5 Academic Center for General Practice, Department of Public Health and Primary Care, KU Leuven, Leuven, Belgium; Faculty of Health Sciences - Universidade da Beira Interior, PORTUGAL

## Abstract

**Background:**

It is particularly difficult for healthcare providers to deliver optimal medical care to multimorbid middle-aged persons because patients’ professional activities, family lives, and other everyday responsibilities hinder them from making necessary lifestyle changes. Our aim was to find out how patients and healthcare providers view and manage the problems of dealing with multimorbidity in middle age.

**Methods and findings:**

This qualitative study consisted of three steps. First, we conducted semi-structured in-depth interviews with 15 purposively sampled middle-aged persons living with multimorbidity to explore the experiences of care in the context of their leisure time, family lives, and work. Second, further individual interviews were carried out to find out the views of 14 healthcare providers. Third, the results of the interviews with patients and healthcare providers were presented to and discussed with four healthcare providers at an interprofessional workshop. Interview data was coded using an inductive-deductive approach and analyzed using content analysis. While patients reflected on challenges in several life domains, healthcare providers differentiated between levels of challenges. Both shared recommendations for better care including i) helping patients cope, ii) providing relief in activities of daily living, iii) continuity of care, iv) interprofessional cooperation, v) health promotion/prevention, vi) expansion of health services and vii) general system-level changes. Furthermore, the healthcare provider workshop highlighted the importance of increasing patient-centeredness, reducing complexity through a care coordinator and promoting interprofessional cooperation/networking.

**Conclusions:**

To further improve the care of patients living with multimorbidity, barriers to managing multiple chronic conditions and facilitators to navigating complex care scenarios should be explored not only for people beyond working age, but for individuals in their mid-life specifically.

## Introduction

Multimorbidity, defined as the co-occurrence of multiple chronic diseases in single individuals [1], presents one of the major challenges for healthcare systems [2]. Although the phenomenon of multimorbidity is often associated with old age, still, over 50% of patients with multimorbidiy are under 65 years of age [3]. While there is no common definition for the middle-age, it is generally considered to mark the phase between professional establishment and retirement [4]. This phase is particularly characterized by multiple and competing role demands with regard to work, family and other life domains [5]. At the same time, the first chronic conditions occur and become increasingly prevalent in midlife [6]. A cross-sectional study found that by the age of 50 years, 50% have at least one chronic condition [7]. Persons with multimorbidity receive complex care, with their self-management requiring time and presenting a financial, but also organizational, social, and emotional burden on both those affected and those that surround them [8]. Especially in middle-aged persons, the management of multimorbidity has a significant influence on their family and leisure activities, as well as work and career [5]. It has been reported, that middle-aged persons with multimorbidity and multiple life demands are at high risk for social isolation and lower health-related quality of life [5, 9]. Furthermore, it has been reported that younger populations experience a higher treatment burden [10, 11]. Vice versa, family life, workplace and free-time activities can impact on the resources of an individual to cope with multimorbidity.

Interventions should be anchored in all life domains affected by care. Family, leisure, and work, as well as points of contact with the healthcare system, should therefore all be taken into consideration [12].

However, research into patients’ and healthcare providers’ views on dealing with multimorbidity in middle-aged persons is limited. While a recent systematic review [13] found an increasing number of qualitative studies focusing on multimorbidity over the last two decades, they often focus on older patients or include a general population, without investigating special needs for the middle aged. Furthermore, those studies were all conducted with general practitioners, therefore not examining views of different healthcare providers. The purpose of our research was therefore to describe how such multimorbid persons experience the management of their multiple diseases in the context of their individual leisure time, their families, and their work. To help in the identification of challenges and the development of recommendations, we also included the views of key healthcare providers involved in their care. This study is part of the MuMiA project (**Mu**ltimorbidity in the **mi**ddle-**a**ged), which aims to improve care for persons with multimorbidity in the 30–60 age group.

## Methods

### The MuMiA-project

The MuMiA project is a mixed-methods study that combines a systematic literature review with qualitative research involving multimorbid middle-aged persons and their key healthcare providers. The results of the systematic review have been published elsewhere [12, 14]. This paper presents the results of a qualitative study conducted in accordance with the consolidated criteria for reporting qualitative research [15] (S1 File). We have described the methodology in detail in a study protocol [16]. The MuMiA study was approved by the Ethics Committee of Goethe-University Frankfurt (Ref. 2021–47, Date: 15.02.2021).

### Study design

A qualitative study design was used to investigate the views of middle-aged persons with multimorbidity, and healthcare providers. In a first step, (a) qualitative interviews with middle-aged persons were conducted to investigate their experiences in managing multiple chronic diseases in the context of their everyday lives. In a second step, (b) key healthcare providers identified by patients during the patient interviews were asked about the challenges they and their patients faced, and their recommendations on how to improve care in this patient population. Results from (a) and (b) were presented to and discussed with healthcare providers at an interprofessional workshop.

### Interview participant selection and recruitment

For the recruitment of participants, information flyers were distributed in general practitioner (GP), orthopedic, rheumatologic and physiotherapy practices, pharmacies, and via self-help groups. Interested persons contacted the study team, whereupon a member of the study team (RB, TSD or MSB) provided detailed information on the study. If they agreed to participate, an appointment for an interview was made. We aimed to include a heterogeneous sample and therefore selected participants using purposive sampling techniques [17]. Persons were included if they i) self-reported having two or more chronic diseases and ii) were between 30 and 60 years old. Prior to the interviews, participants were required to provide written informed consent and a short questionnaire on sociodemographic characteristics, family, and work. Data on race/ethnicity were not collected.

To select types of healthcare providers for the study, we asked participating multimorbid patients for key healthcare providers involved in their care. These included GPs, physicians from other specialties (i.e., cardiologists, orthopedic surgeons, endocrinologists, ophthalmologists, neurologists), pharmacists and physiotherapists. GPs were recruited from *ForN*, a practice-based research network in primary care [18, 19]. The other healthcare providers were recruited based on institutional or personal contacts. One member of the study team (RB) provided detailed information on the study, and interested parties were included if they were involved in the care of multimorbid persons between 30 and 60 years old. Prior to the interviews, healthcare providers were required to provide written informed consent and to fill in a short questionnaire on their sociodemographic characteristics and details on their professional environment.

### Interview data collection

Individual interviews were conducted by one researcher (CS, JVS, RB, TSD or MSB) based on a semi-structured interview guide (S2 and S3 Files). Stakeholder-specific interview guides were developed according to the manual for conducting qualitative research by Helfferich [20] (one for patient and healthcare provider interviews each) based on the results of the previous systematic review [12]. They were discussed at an interprofessional qualitative research workshop at the Institute of General Practice at Goethe University Frankfurt and were pilot tested on two patients and one healthcare provider. Interviews were conducted between April and September 2021 via telephone using “DFNconf”, a secure conference tool provided by the “German Research Network”. Interviews were recorded with the consent of the participants and transcribed by a professional transcription office. To ensure data protection, results were presented using pseudonyms (“P” for “patient”, “GP” for general practitioner, “SP” for specialized physician, “PC” for pharmacist and “PT” for physiotherapist, an individual number, and “f” for “female” or “m” for “male”). After the interviews, we took field notes. Interviews were not repeated, and transcripts were not returned to patients or healthcare providers for approval.

### Interview data analysis

Interview transcripts were imported into MAXQDA-18 [21], and two researchers (TSD and RB) used qualitative content analysis according to Kuckartz to analyze them [8]. Interviews were coded using a deductive-inductive approach. Deductive codes were derived from the interview guide, whereby inductive codes were added based on the data itself [22, 23]. The two researchers coded the interviews independently and new categories added by inductive coding were agreed upon. Separate coding trees were developed for patient and healthcare provider interviews. The interviews were conducted in German and selected quotes were translated into English by a native speaker.

### Workshop

After the interviews had been analyzed, participating healthcare providers were invited to take part in an interprofessional workshop. The workshop was guided by an external moderator (CG) and was held online due to the COVID-19 pandemic, using the Zoom conference software system. The purpose of the workshop was to discuss and interpret results (triangulation), and to identify recommendations on how to better provide care for middle-aged multimorbid patients. Protocol notes were taken from the discussion and the results were documented and evaluated by three researchers (TSD, RB, MVDA).

### Researcher characteristics

As researchers’ assumptions and interests may have influenced the analysis and interpretation, selected characteristics are also provided: TSD (female): health scientist and researcher; RB (male): pharmacist and researcher; CS (female): researcher and physician; MSB (female): physiotherapist and researcher; MD (female): health scientist and researcher; AIGG (female): general practitioner, health manager and researcher; MVDA (female): health scientist, researcher, and professor of polypharmacy and health services research. Characteristics of further involved researchers: JVS (female): student; CG (female): psychologist and senior researcher.

## Results

### Participants

A total of 29 participants completed the qualitative interviews, of whom 15 were patients and 14 healthcare providers. Of the healthcare providers, four agreed to participate in the subsequent interprofessional workshop (1 GP, 2 pharmacists, 1 physiotherapist). The average interview duration was 38 minutes (range: 13–80 minutes).

Tables 1 and 2 summarize the key characteristics of participating patients and health care professionals, respectively. On average, patients had four (range 2 to 10) chronic diseases and reported to take four (range 1 to 8) medications per day including OTC. The most prevalent conditions in our sample were cardiovascular disease (n = 10, 67%), eight (53%) had musculoskeletal disorders, seven (47%) had a disease of the thyroid gland and seven (47%) had symptoms of depression. Furthermore, the included participants had a wide range of other chronic diseases, including diabetes mellitus, atopic dermatitis, asthma, restless legs syndrome, obesity, and irritable bowel syndrome. All patients were physically impaired, while six (40%) had at least one additional mental disease.

**Table 1:** Characteristics of participating middle-aged persons with multimorbidity (n = 15).

**Age (years)**	Mean	50
	Range	31–59
**Sex, n**	Male	4/15
	Female	11/15
**Educational level, n**	Tenth grade secondary school graduation certificate	5/15
	University entrance certificate (after 12–13 years of school), or equivalent	9/15
	Not reported	1/15
**Employment, n**	Yes	9/15
	No	3/15
	Seeking work	2/15
	Sick leave	1/15
**Working hours per week (h)**	Mean	30
	Range	10–45
	Not applicable (n)	4
**Marital status, n**	Single	6/15
	Married	8/15
	Divorced	1/15
**Children, n**	Yes	8/15
	No	7/15

**Table 2:** Characteristics of participating healthcare providers (n = 14).

**Age (years)**	Mean	48
	Range	28–61
	Not reported (n)	1
**Sex, n**	Male	4/14
	Female	10/14
**Years of experience (years)**	Mean	20
	Range	3–34
	Not reported (n)	1
**Type of employment, n**	Self-employed	7/14
	Employed	6/14
	Not reported	1/14
**Occupation, n**	General practitioner	8/14
	Psychiatrist	1/14
	Neurologist	1/14
	Pharmacist	2/14
	Physiotherapist	2/14
**Number of patients, n**	Mean	150
	Range	20–350
	Not reported (n)	1
**Proportion of multimorbid patients**	Mean	26
	Range	5–70
	Not reported (n)	1

### Interviews

During the interviews, both patients’ and healthcare providers’ reflected on perceived challenges related to the care of patients with multimorbidity. While patients’ reflection focused on several life domains, healthcare providers differentiated between levels of challenges. Table 3 presents the key challenges described by each group.

**Table 3:** Challenges identified by middle-aged persons and their healthcare providers.

Interview	Key challenge	Examples
**Patients**	**General consequences of multimorbidity**	• Symptoms • Drug-related side-effects and interactions • Psychological stress • Shame and stigmatization, lack of awareness and understanding • Burden of treatment and self-management
	**Impact on daily life**	• Limitations to basic and instrumental activities of daily living • Accessibility • Scheduling rather than spontaneity
	**Impact on leisure time**	• Limitations relating to social activities • Limitations relating to ability to travel • Limitations relating to physical exercise • Limitations relating to hobbies
	**Impact on work / career**	• Decreased work ability/productivity • Low concentration due to medical side effects • Lack of understanding and consideration by colleagues • Reduced working hours and income • Compatibility of work and medical appointments
**Healthcare providers**	**Patient level**	• Multiple stressors (family, work) • Multimorbidity as a burden that middle-aged people have little time to deal with Lifestyle changes = main challenge (little awareness, low motivation) • Limited time available for preventive and health promoting activities • Unrealistic expectations concerning capabilities, little acceptance of the diseases • Feelings of shame • Lack of interest in attending check-up appointments, low adherence to therapy
	**Healthcare provider level**	• Lack of continuity of care (long waiting times for specialists) • Lack of interprofessional communication and cooperation • Lack of care coordinator • Consideration of patient preferences sometimes difficult • Lack of time (for consultation/collaboration with colleagues)
	**System level**	• Little high-quality patient information available • Existing services do not match middle-aged patients’ needs • Statutory health insurance provides limited coverage of health services • Focus on short-term cost savings rather than long-term prevention

#### Challenges identified by middle-aged persons with multimorbidity

*General consequences of multimorbidity*. Participants described a number of health-related consequences of living with their chronic diseases. These included, for example, direct symptoms of their specific diseases, as well as drug-related side effects and interactions (e.g., diarrhea, lack of concentration, weight gain, urinary retention, and gastrointestinal problems).

More than half the participants said that suffering from multiple chronic diseases led to psychological stress, e.g., related to accepting their diseases and the resulting consequences, such as limited mobility or uncertainties concerning family planning:

“*Whether to dare to go through pregnancy was a big topic for me*, *especially because of my scoliosis*. *[…]*. *And would it even be possible*, *or would it put too much strain on my spine etc*. *[…] and you’re afraid when you have [a disease]*: *Have I passed on the genes to my children or not*?*” (P04f)*

In addition, while some patients were embarrassed that their diseases were visible to outsiders, others were pleased that their diseases were not easily recognizable:

“*It’s often not taken seriously*, *is it*? *Because you don’t often recognize it in people*, *no matter what chronic illness they have*.*” (P05f)*

Participants also spoke of feelings of shame and stigmatization as a result of their diseases:

“…*I use three different insulins a day*, *and in the beginning*, *for example*, *I was ashamed to take a [insulin] shot in public because it was kind of–yes*, *it looked a bit sleazy*, *a bit junkie-like*.*” (P03f)*

Another problem mentioned by participants was the general lack of knowledge about their diseases, as it led to a lack of both understanding and awareness:

“*And then there’s this general knowledge*, *‘Oh*, *you have [diabetes]*, *well*, *then you’re not allowed to eat cake at all’*. *And then I freak out on the inside*. *[…] If you have an illness that everyone thinks they know about*, *then you get stigmatized a bit*, *right*? *That’s–yes*, *so you feel like a bit of an alien*.*” (P03f)*

Many interviewees described the difficulties involved in having several diseases and treatments simultaneously. These included, for example, the coordination and integration of many doctor’s appointments into their everyday lives.

Participants also spoke of being overwhelmed by interactions between their individual illnesses and having to take several medications:

“… *I’m barely awake*, *I’ve just opened my eyes and already have to inject myself with ten units of insulin*. *And then after that I have to swallow another five pills before doing anything at all*, *before I can even begin the day*.*” (P03f)*

*Impact on daily life*. Participants further described the limitations to basic activities of daily living that resulted from their diseases. In this context, difficulties with functional mobility, including climbing stairs, sitting, and carrying things, as well as bathing were mentioned.

Participants also described the challenges they faced performing such instrumental activities of daily living as preparing meals, shopping for groceries, cleaning and other household chores, as well as driving a car and taking care of pets.

Most patients described their diseases as running *“like a thread throughout the day*” *(P03f)* and explained how they take account of their illnesses when planning their everyday activities:

“…*I take my medication early in the morning*, *but then I’m limited for a few hours in that I have to refrain from doing certain tasks and from some movements […] The practical upshot of that is that I postpone my work and things to later in the day*.*” (P14f)*

*Impact on leisure time*. Participants described challenges in planning and carrying out leisure activities, adding that these limited their participation:

“… *when friends want to do something*, *I always have to think first*: *Can I do that*? *Because of my weight*, *because of my bones*, *because of my mental illnesses–will I manage*? *Is it even possible for me*, *do I really want to*?*” (P08f)*

Regarding travel, the avoidance of long flights was described as a consequence. The secure transport and storage of medication (e.g., the possibility of cooling insulin), and the availability of medical care while travelling were also considered relevant.

Often as a result of their physical limitations, physical exercise and pursuing hobbies were a challenge for most patients, and were only possible after extensive preparations and / or to a limited extent.

*Impact on working lives*. Almost all interviewees mentioned the negative impact their diseases had on their professional activities, such as difficulties carrying out tasks that were part of their everyday work. Besides the physical effort of such activities, this included, for example, attending meetings and getting to work. Participants also complained of pain and concentration disorders, which tended to hamper their performance.

As a result of their limited resilience, participants also mentioned concerns about job security as well as stigmatization from their colleagues because *“*… *no real thought is given to whether you are actually ill*.*” (P07m)*.

A limited ability to work as productively as before often resulted in a reduction in working hours or the inability to work at all. Although they appreciated being paid per hour or being able to work part-time, loss of income was a problem.

One person described that it was difficult to reconcile medical appointments and professional activities:

“*And sometimes I feel a little uncomfortable with having to say*, *‘I’ll be gone the rest of the day because I have to look after my health’*.*” (P03f)*

#### Challenges perceived by healthcare providers

*Patient level*. From the perspective of healthcare providers, multiple commitments in different areas of their middle-aged multimorbid patients’ lives meant they faced a range of demands and stressors. In addition to professional activities, responsibilities in their private and family lives, such as caring for children and parents, were also mentioned. Due to these various obligations, providers said their patients lacked time, which made the management and reconciliation of their health-related appointments and everyday lives extremely difficult:

"…*I think that the middle-aged patient is often stuck in a sandwich*, *that they may have to take care of their parents*, *that they have children*, *that they have jobs—that there are just so many things going on in their everyday lives that they—and I actually see that as more extreme in women than in men–forget their own needs completely*.*” (GP07f)*

Healthcare providers said one of the main challenges they faced was convincing patients of the need to change their lifestyles. On the one hand, this reflected a lack of awareness, but also little motivation to change their habits. On the other hand, they saw that patients didn’t have the time for health-promoting and preventive activities such as physical exercise:

“… *encouraging patients*, *or getting them to understand that there are other things they could do to avoid being dependent on pills*, *such as lose weight or play sports or avoid certain foods*. *Of course that is often very difficult to convey*, *because of course it interferes with their lifestyle*, *and very few people are willing to accept that*.*" (GP01f)*

As a special characteristic of this group of patients, providers described the enormous demands patients made of themselves, which made it difficult for them to accept their illnesses:

"*Higher*, *faster*, *further [*…*] The demands they make of themselves are insane and could hardly be met by someone in good health*.*" (SP13f)*

In connection with acceptance, one caregiver also said patients often reacted negatively to the prospect of having to use assistive devices:

"*A diagnosis of MS is not severe [for patients]*. *Well it makes them suffer too*, *but somehow they manage*. *But if I say they need a wheelchair they collapse in a heap*.*” (SP13f)*

Healthcare providers also said their work was made difficult by the fact that patients often lack any understanding of how the disease develops and of its potential long-term consequences, adding that this was reflected in negligence and a low level of initiative with respect to attending check-up appointments:

"*That’s actually the most difficult thing of all*, *making them understand that it’s a question of their health in the next ten*, *twenty years that’s at stake*, *and that that’s the reason the doctor checks things*.*" (GP01f)*

*Healthcare provider level*. Some respondents described working with other providers as difficult, often due to a lack of time but also a *“lack of collegial behavior” (GP01f)*, which made cooperation with other providers challenging:

"… *it would be very nice if it could be done interdisciplinarily*, *and physicians would work with other therapists*, *or with pharmacists on drug therapies*. *Unfortunately*, *due to time constraints*, *I don’t think it’ll happen*.*" (PT02m)*

Another problem the providers identified was discontinuity, caused, for example, by long waiting times to see specialists when the responsible provider is on vacation, or by poor accessibility in rural areas:

“*In rural areas there’s often the problem that specialists are difficult to reach […]*. *And there are often real gaps in care because practices obviously cannot guarantee that people will be able to get hold of their family doctor within half a day or a day if something is urgent*.*” (GP09f)*

In addition, continuity of care cannot be guaranteed because of a lack of coordination:

"… *many people in the age group up to around 50 or so don’t even have a permanent general practitioner*, *so that when they have health issues*, *they just go to the physician they think is the right one*. *[…]*. *And when they go somewhere*, *it is not reported anywhere*, *and there is no coordination*.*" (GP09f)*

There is also a lack of information continuity in existing cooperation, e.g., reports are not completed properly or only skimmed over, which sometimes results in such errors as duplicate prescriptions and inappropriate medication:

"*Then they get*, *I don’t know*, *let’s say Diclo[fenac] 75 twice [daily] from the specialist*, *and I would throw my hands up in the air because of their reduced kidney function*. *Well*, *and then I have to be careful about saying it because I don’t want to criticize my colleagues or reduce the patient’s confidence in the doctor*, *but obviously I don’t want the kidneys to fail either*.*" (GP07f)*

Sometimes providers also found it challenging to reconcile patients’ preferences with their therapy, e.g., the desire to have children.

*System level*. One provider pointed out that the provided information was difficult for patients to understand. In her opinion, the extensive and unfiltered information leads to uncertainty and undesirable behavior on the part of patients:

“[…] *they adjust their medication themselves*, *for example*, *or take some dietary supplements and no longer consult their physician […]*. *There is actually too much information and patients*, *as lay persons*, *really can’t judge what is of use to them*.*" (GP09f)*

At a structural level, criticism was also leveled at the fact that existing services did not address the needs of middle-aged people:

*"There are sometimes few opportunities for the middle-aged population because only groups for the old exist*, *in which geriatric sports are on offer, and not really at an intensity that would be suitable for middle-aged persons*.*" (GP05m)*

In addition, budgets available to providers, and the scope of therapies covered by health insurance companies, were regarded as inadequate. The focus on short-term treatments rather than investing in prevention has also been criticized:

“*It’s always about cost savings; so the healthcare system is always threatened by cost cutting and is always seen as driving costs*. *But that is a very short-term perspective because the costs that I save now*, *this year […] often [re]appear as significantly higher secondary costs in old age*.*" (PC11f)*

#### Recommendations

During the interviews, both groups of participants had ideas and recommended approaches that might enable existing care challenges to be met, whereby patients generally described how they dealt with everyday multimorbidity, and providers focused on concrete recommendations. Although patients and healthcare providers were interviewed independently, they often had the same ideas, or their ideas complemented one another. The results of both patient and healthcare provider interviews are therefore presented together in Table 4.

**Table 4:** Patients’ and healthcare providers’ (HCP) views on how to improve the healthcare situation of middle-aged persons with multimorbidity.

**1. Support patients in coping with diseases**	
Patients	• Obtain information and understand diseases and their interrelationships • Information materials targeted to the needs of middle-aged persons • Expansion of self-help groups for specific diseases
HCPs	• Educate and motivate patients • Promote self-care and self-management • Bring patients together, e.g., in self-help groups • Prepare a “patient guide” that explains the healthcare system and the organization of healthcare-related activities
**2. Relief in daily life**	
Patients	• Support in activities of daily living, e.g., through social support or paid services • Support professional environment (understanding)
HCPs	• Support in the care of children and older family members • Promote participation of people with disabilities • Compatibility of work and healthcare-related activities • Financial relief for patients
**3. Continuity of care**	
Patients	• Shorter waiting times for specialist appointments • Individual and holistic therapy plans
HCPs	• Care coordinator • Disease management programs • Timely appointments, e.g., in acute cases and hospitalization • Continuous medication supply, e.g., supported by pharmacists
**4. Interprofessional cooperation**	
Patients	• Communication and cooperation between healthcare providers
HCPs	• Communication and cooperation between healthcare providers • Agree on common therapy goals • Networking • New educational concepts (interprofessional education)
**5. Health promotion and prevention**	
Patients	• Raise awareness and increase knowledge
HCPs	• Education of patients • Improve information materials • Set up advisory centers • Anchor health promoting behavior in schools
**6. Expansion of health services**	
Patients	-
HCPs	• Extend preventive services and adapt them to suit patients’ needs • Extend mental health care services
**7. General system-level changes**	
Patients	-
HCPs	• Lower bureaucratic obstacles • Defragment the healthcare system • Provide financial incentives and sanctions (higher taxes, risk-specific fees) • Make patients’ medical data centrally available • Electronic prescriptions

*Help patients cope with diseases*. To better cope with their diseases, patients often seek information by, for example, reading or watching videos. In this context, patients wanted available information and training courses to be attuned to their specific situation, for example with respect to medication, nutrition, stress management, motivation, and information materials.

Providers saw it as their responsibility to educate and motivate patients, and to help patients improve and maintain their health by supporting and empowering them to rely more on self-care and self-management.

“… *in any case*, *it is always very important to clarify that the patient is old enough to deal with it himself*. *And that he is being given the opportunity to live in such a way that he is able*, *taking the example of diabetes*, *to eat and change his lifestyle in such a way that it improves his health as much as possible*.*” (PC12f)*

To help patients cope with their diseases, providers saw clear advantages in bringing patients together, e.g., in self-help groups. One healthcare provider explained they *“…need to discuss things with others that have been affected*, *with like-minded people*.*" (PC11f)*. Although self-help groups were also welcomed by patients, the amount of groups were not perceived as sufficient.

Regarding treatment burden, providers also acknowledged that the high complexity and fragmentation of the healthcare system might be too much for patients to deal with. Providers therefore thought a guide or contact person for patients whose main function was to help patients organize care-related activities and help them “*find their way around the healthcare system more easily*, *to get to the providers they need*” *(PC11f)*.

*Relief in daily life*. Regarding basic and instrumental activities of daily living, many multimorbid persons considered their friends and family to be the main source of support, while others had good experiences with services such as paid help in the household, cleaning staff and grocery delivery services. Healthcare providers added that patients should receive support in the care of (older) relatives and children and that the cost of such services be covered by health insurers.

Activities of daily living also include participation in the community and society. With regard to people living with disabilities, participants saw potential for improvement in the infrastructure:

“… *just needs to be generally made a bit more accessible*, *right*? *That sidewalks*, *entrances to public places basically just have to be made more barrier-free*.*" (PT14f)*

To further support patients whose ability to work and earn money was limited by their disease(s), providers suggested providing financial relief to patients. In order to increase the compatibility of diseases and work, providers suggested that employers could give patients the opportunity to adjust their working hours to take account of their medical appointments without any disadvantage to them.

*Continuity of care*. To improve the health of middle-aged patients with multimorbidity, both patients and healthcare providers recommended improving continuity of care. Middle-aged persons with multimorbidity wished for shorter waiting times for specialist appointments, and for an individual and holistic therapy plan. Providers felt that continuity of care could be achieved by ensuring one provider (e.g., general practitioner or pharmacist) coordinated healthcare, especially when several providers or sectors are involved in the treatment:

"*I think patient safety is always higher when care is continuously and regularly provided in a general practice that also keeps an overview of things*.*" (GP06f)*

The structure of disease management programs (DMP) was considered to be particularly helpful in this regard. Providers further wanted appointments to be scheduled more quickly, especially when patients had acute problems. They thought that in order to provide appropriate care, specialist appointments and hospital admissions should be arranged faster. Continuity of care was also highlighted with respect to a continuous supply of long-term medication, which is sometimes interrupted because the GP is on vacation.

"…*that long-term medication could be provided straight away*, *via the pharmacy*, *at least for a while [*…*]*. *There should be some kind of arrangement so that long-term medication can really be taken continuously without there always being the problem that someone somewhere is closed at the moment or is on vacation*.*" (PC12f)*

*Interprofessional cooperation*. Both patients and providers considered communication and cooperation between the providers involved in their care to be extremely important. This was not confined to passing on information, but also to formulating a common therapy goal and to cooperating on an equal footing:

"… *Handover protocols and simply agreeing with each other now and then on how things are going*, *what’s going on*. *I think it would be best if all the therapists*, *but also the doctors*, *discuss it among themselves and fix a direction to go in*.*" (PT02m)*

Providers thought it was important that networking should involve representatives from other institutions and sectors than their own. They also thought quality circles might help in this respect. To further promote interprofessional cooperation over the long term, it was also considered important to train medical and pharmacy students in one group, and to implement interprofessional education concepts.

*Health promotion and prevention*. Key aspects that were mentioned during the interviews included activities to promote health in the general population. This could be carried out by improving health education and using more preventive therapies, with the overall aim of raising awareness and increasing knowledge. In this context, patients hoped this would generate more understanding for their diseases.

Providers considered education and information for patients to be important, and saw potential to improve patient information, for example by providing patients with information that was targeted according to their needs, and in a succinct, easy-to-understand way.

"*I think that factual*, *easy-to-understand information on the whole clinical picture is needed*, *along with a factual*, *not frightening*, *evaluation of possible consequences of the disease*. *Everything should be short and concise*.*” (PC11f)*

To distribute information more effectively, they saw potential in using social media, information events and setting up information/counselling centers, and to anchor health promoting behavior, providers suggested that prevention should become part of school education.

*Expansion of health services*. Further recommendations included expanding health services. On the one hand, these concerned the necessity of preventive measures such as courses to prevent back pain, high blood pressure, and measures to help patients stop smoking. From the perspective of healthcare providers, existing services should specifically target middle-aged patients, and should be permanently available.

"… *what I think we’d find helpful is nutritional advice*, *in addition to a structured sports program or activity program*, *meaning a program that has been adapted to meet the needs of the patients*.*” (SP08f)*

On the other hand, both middle-aged persons with multimorbidity and healthcare providers saw a need for more psychological care for patients. Providers explained that basic mental health care and other health services should be low-threshold, easily accessible, and available free of charge.

*General system-level changes*. To improve the care of multimorbid, middle-aged patients and patients in general, providers also suggested making broad changes to the healthcare system, such as lowering bureaucratic obstacles.

"… *if [a therapy] first requires an expert opinion and then approval from the health insurance company and then perhaps needs to be adapted by the provider of an assistive device [*…*] then motivation disappears relatively quickly*.*" (PC11f)*

The necessity of defragmenting the healthcare system was also mentioned, since patients with multimorbidity receive care from multiple healthcare providers and across many interfaces.

Providers saw potential in increasing motivation through the use of financial incentives. As an example, they mentioned that although DMPs were considered useful, the enrollment of patients in several DMPs was unattractive financially.

Interdisciplinary cooperation must also be *"promoted in terms of time and money" (PT02m)* and the review of medication plans by pharmacists should be expanded and rewarded accordingly.

Increased financial compensation would also help ensure that caregivers receive adequate payment for their care services and that more time is available for care

"*It takes an awful lot of time to go through the medication plan again and maybe change it*, *and then explain everything again*. *[…] Yes*, *and with multimorbid persons there are simply a load of things you have to consider*. *[…] And of course*, *that is not reflected in our pay*.*” (GP10f)*

Regarding preventive measures, measures such as higher taxes for salt and sugar, and risk-specific contributions to health insurers were also discussed.

Healthcare providers also hope the digitization of processes will improve healthcare. The focus here was on making patient data related to medications, findings, and reports centrally available in an electronic patient file. This would improve continuity of care, especially at care interfaces. Furthermore, the e-prescription was regarded positively.

### Workshop results

The results of both patient and healthcare provider interviews were presented to and discussed by healthcare providers at an interprofessional workshop. The healthcare providers then transformed the ideas into key recommendations on how to improve the healthcare of middle-aged patients with multimorbidity.

#### 1. Increasing patient-centeredness

In view of the numerous demands made on middle-aged persons, healthcare providers thought the demand for patient-centeredness should entail viewing the patient in the context of his or her life domains. Providers saw a necessary trade-off between the ideal therapy and feasibility in the everyday lives of this patient population. While their general aim was to help patients to cope with their diseases, to promote self-care, self-management, and healthy behaviors, and to focus on patients’ mental health, providers also suggested that structural arrangements and digital solutions would help patients flexibly integrate their health care into their everyday lives. Such measures might include, for example, longer opening hours, or even offering consultations that are independent of working hours and could take place online. In this context, the use of e-prescriptions was also encouraged.

#### 2. Having a care coordinator to reduce complexity

The complexity and fragmentation of the healthcare system hinder continuity of care and result in medication errors such as duplicate prescriptions and drug-drug interactions. A care coordinator could guide patients and help them oversee and coordinate their health-related activities. According to providers, this could be achieved by strengthening the role of GPs, by involving pharmacists, or by creating a new position. Overall, they agreed that providers should be compensated for coordinating patients’ care.

#### 3. Promote interprofessional cooperation and networking

Although appreciated by healthcare providers, interdisciplinary and interprofessional cooperation is currently barely feasible due to time constraints and a lack of the necessary structures. Providers said it would only be possible in their leisure time if personal relations existed, or if providers lived close to one another. Providers therefore recommended implementing structures that support interprofessional cooperation, and enable providers to get to know and trust each other (e.g., joint education and training). Furthermore, multiple providers involved in the care of multimorbid persons should agree on common therapy goals and jointly develop treatment plans to improve patients’ health and avoid contradictory statements and treatments. For this special patient population in particular, providers recommended multimodal treatment plans involving multiple healthcare providers. Cooperation between pharmacists and GPs was additionally considered necessary to enable an exchange of pharmacotherapy-related information for patients with polypharmacy. Overall, digitization and in particular electronic patient files were expected to facilitate interprofessional cooperation.

## Discussion

The results of the patient and healthcare provider interviews showed the wide range of challenges faced by those living (as patients) or providing care for people (as healthcare providers) with multimorbidity in middle age. Both patients and healthcare providers saw the difficulties for this patient population of looking after their health without experiencing functional losses in their life domains. From the perspective of patients, multimorbidity had both physical and psychological consequences that also had an impact on their everyday and professional lives. During the interviews, it became clear that most patients suffered more from the limitations caused by one index disease than from multimorbidity itself. The presence of, or interaction between, multiple chronic conditions was rarely addressed by patients. Mental health was a central issue for most patients and was often linked to difficulties coping with and accepting their diseases, and an increase in the psychological stress and burden of treatment and self-management.

Healthcare providers identified challenges on a patient, provider, and system level that prevented middle-aged multimorbid patients from receiving appropriate care. From their point of view, one of the main challenges on a patient level was how to convince patients to focus on health-promoting, preventive, and self-management activities [24] within the context of their multiple life domains and limited time resources. In this context, it is important that healthy lifestyle activities are easy to adopt and the easier and obvious choice for patients [25]. One study showed that health promoting activities can extend life up to 7.6 years for middle-aged patients living with multimorbidity [26]. According to providers, the current healthcare system further fails to deliver adequate care on a healthcare provider level and to address the specific care needs of middle-aged multimorbid patients. Improvement strategies recommended by patients and healthcare providers included increasing patient-centeredness and agreeing on a trade-off between patients’ health care activities and their everyday lives, as well as promoting continuity of care and interprofessional cooperation. The results of our study strengthen recommendations on multimorbidity that have been published by the World Health Organization, including necessary changes on policy and system levels, improvement of care coordination and self-management support, and simplifying treatment regimens [3].

Generally speaking, many of the problems identified in this research are not age-related, but concern multimorbidity in general. Nevertheless, the setting and the resulting implications differ from care for older people with multimorbidity, especially with regard to time constraints, multiple stressors and the prevention of deterioration in conditions.

It is worth mentioning that previous studies focusing on multimorbidity in middle-aged persons differ greatly in their definitions of both multimorbidity and middle age. While another German study [27] used exactly the same criteria for multimorbidity and middle age as we did, a study from Singapore included patients from 40 to 64 years with three or more chronic conditions [9] and a study from Chile defined multimorbidity as the existence of two or more chronic conditions but only investigated women aged between 40 and 59 years [28]. While the questionnaire-based study from Singapore identified an association between multimorbidity and a decrease in certain aspects of health-related quality of life [9], both the study from Germany and one from China discovered that the risk of developing multimorbidity was greater in socially deprived people, which suggests that multimorbidity is avoidable and preventive measures are particularly important [27, 29].

Overall, our findings agree with the results of our recently published systematic review assessing existing literature on middle-aged people living with multimorbidity, which found that their ability to perform activities of daily living or pursue their work was hindered by their diseases [12]. Mental diseases accounted for half of the conditions included in the systematic review, and when they were present, they were associated with greater disability in all life domains. We found one further study [30] that discovered that the number of diseases negatively influenced self-care management and self-maintenance. Rosbach et al described in a systematic review [31] that multimorbid patients use strategies such as prioritizing certain treatments to reduce the workload.

Some members of our research group recently conducted a qualitative study presenting stakeholders’ views on the development and implementation of a polypharmacy management program and found that a lack of care coordination and continuity of care at a healthcare provider level was a major problem [32]. They also emphasized that any intervention in this patient population should be practicable, concrete and feasible in terms of time and effort.

The challenges multimorbid patients face when attempting to integrate their care needs into their everyday activities have been described in previous research, and recommendations on how to improve the situation have been developed. As an example, according to the Ariadne principles, the most important strategy when dealing with patients with multimorbidity is for physicians and patients to share “realistic treatment goals” [33]. In a systematic guideline review of evidence supporting the best clinical management of multimorbidity, Muth et al. emphasized the importance of patients and healthcare providers communicating their respective needs, priorities and preferences when developing a care plan [34]. A scoping review by Valderas et al. also highlighted that especially when dealing with persons with multiple chronic diseases, person-centered care should be a guiding principle, and patients’ values, goals and priorities should shape care delivery in the form of individual care plans [35].

### Strengths

The use of a qualitative study design to answer the study question made it possible to identify different aspects relating to the healthcare situation of middle-aged multimorbid patients. Purposive sampling enabled us to target patients according to their characteristics, e.g., age, gender, type of chronic condition, educational level, employment, and marital status. This is particularly relevant in this context, since patients with multimorbidity represent an extremely heterogeneous group that have complex care needs and have only seldom been targeted by research. As this is one of few studies to concentrate on multimorbidity in middle age, it was possible to gain new insights, and this is one of its strengths. Another strength was the stakeholder approach, and the inclusion of both patients’ and providers’ perspectives. By selecting healthcare providers based on patient interviews, we were also able to make sure we included providers that were considered relevant by patients. Discussing the results from the interviews with providers at the workshop further enabled us to synthesize and prioritize recommendations on how to improve the situation of middle-aged patients with multimorbidity.

### Limitations

However, our study also had some limitations, for example with respect to the distribution of gender and age, as the vast majority was female, and more than half the participants were in their 50s. This may have led to an underrepresentation of certain topics that specifically concerned people in their 30s and 40s, such as the care of young children, older relatives and career building. In addition, data on race/ethnicity was not collected which hindered us from reflecting our results in this context. Furthermore, the decision on which healthcare providers to include in our study was based on patient interviews. While the enumeration of relevant providers by patients was broad, this approach might nevertheless have led to the non-consideration of potentially relevant healthcare providers. Although mentioned by patients, we could not consider dentists, as we were unable to recruit any that felt confident talking about dealing with multimorbid patients, and we did not include providers that practiced alternative medicine. Last but not least, the issues related to pay-for-performance and the healthcare system may be specific to Germany which hinders the transferability of results to other health systems with different organizational and financial models.

### Conclusions

According to our research, both patients and healthcare providers are aware of existing challenges and how to address them. However, it is not feasible for them to overcome these difficulties on their own. This suggests that general systemic change is needed. Policymakers should strengthen the healthcare system by lowering bureaucratic obstacles, making more financial resources available, promoting digitization and continuity of care, moving from disease- to patient-centeredness, and not least by promoting interprofessional cooperation and coordination of care. Further research should focus on how to implement these ideas and on identifying what preconditions must be fulfilled before they can function in a way that is beneficial to patients.

## Supporting information

S1 File Consolidated criteria for reporting qualitative research (COREQ) 32‐ITEM checklist. (PDF)

S2 File Interview guides. (DOCX)

S3 File Interview guide HCP. (DOCX)
