# Supplementary material for: How do middle-aged patients and their healthcare providers manage multimorbidity? Results of a qualitative study
Source: PLoS One. 2023 Aug 31;18(8):e0291065. doi: 10.1371/journal.pone.0291065 (PMC10470875; doi:10.1371/journal.pone.0291065)
Supplement: S2 File — (DOCX) [file pone.0291065.s002.DOCX]

| **ID interview partner** |  |
| --- | --- |
| **Interviewer** |  |
| **Date and time** |  |
| **Interview duration** |  |

**Introduction to interview**

– prior to interview: appointment has been made and a consent form has been signed *–*

- Greet the interview partner, introduce yourself, thank for participation
- **Introduce project:**

*Good morning/afternoon Mr./Mrs. XXX,*

*Thank you for participating and supporting our research project. My name is XXX and I am a researcher at the Institute of General Practice at Goethe University. We are conducting the interview as part of our MuMiA study, the aim of which is to describe the everyday lives of patients with several chronic diseases, the limitations they face, and the strategies they use to overcome them. Based on these interviews, we would like to find out about the everyday problems and stress involved in dealing with the diseases in order to develop recommendations on how they could be alleviated. The project is being financed by a charitable foundation entitled* Stiftung Gesundheitswissen *(health knowledge). Everything you say will be treated confidentially and anonymously. Our discussion will last approximsately 30-40 minutes. We are interested in what you think, which is why we consider you to be an expert. There are no wrong answers. If you don’t object, I would like to record our discussion.*

- Start the recording once you have the interviewee’s consent
- Participation is voluntary
- Data protection – no personal details will be published
- Point out that at the end of the interview, the interviewee will again be asked if all comments may be taken into consideration in the study
- Are there any questions about the procedure?

**The interview**

| **Topic** | **Sub-topic** | **Questions** | **Further questions/reminders** |
| --- | --- | --- | --- |
| First group of topics: **Introduction and daily routine** | Problems/ Stress factors | Please tell us in what situations or at what times in your everyday life you become aware that you have to deal with several diseases simultaneously? | -How does that differ from the situation when you had one/fewer diseases to deal with?  - Please describe stressful situations you have experienced due to interactions between your diseases. |
|  | Resources/ Strategies to overcome difficulty | -In your everyday life, what helps you deal with the problems you have described? | -What helps you?  -Who helps you?  -What doesn’t help?  -What helps compensate?  -What would you wish for? |
| First group of topics, part a: **profession/career** | Problems/ Stress factors | Please describe stressful situations that you experience in your working life as a result of your diseases. | -How have your diseases affected your career?  -Do you think your diseases have influenced your career? |
|  | Resources/ Strategies used to deal with them | -What do you rely on in order to deal with the problems you have described? | -What helps you?  -Who helps you?  -What doesn’t help?  -How do you feel about that?  -How do you deal with the situation? |
| First group of topics, part b: **Leisure time/free time** | Problems/ Stress factors | Please describe stressful situations that you experience in your free time as a result of your diseases. | Are there activities that you can’t participate in, or from which you are excluded, for health reasons? |
|  | Resources/ Strategies used to deal with them | -What do you rely on in order to deal with the problems you have described? | -What helps you?  -Who helps you?  -What doesn’t help?  -How do you feel about that?  -How do you deal with the situation? |
| First group of topics, part c:  **Social network (Family/ Friends/ Neighbors)** | Problems/ Stress factors | Please describe stressful situations and or limitations that you experience in your everyday life that involve your family and/or your friends and result from your diseases. | -Are there activities that you can’t participate in for health reasons?  -What influence do your diseases have on your family/social environment?  How do you deal with that? |
|  | Resources/ Strategies used to deal with them | -What helps you deal with the problems you have described? | -What helps you?  -Who helps you?  -What doesn’t help? |
| Recommendations for improvement | Prevention | -What measures help you maintain or improve the state of your health?  -What obstacles can you overcome with the help of others or by using your own initiative?  What would be necessary in order to help others with similar limitations? | What measures to maintain or promote your health would you like to take advantage of? |
|  | Reducing obstacles |  |  |
|  | What would be necessary to achieve this? |  |  |
| Support in self-management | Ideas | Please describe to us what you do when confronted with difficulties, stress and obstacles? How do you deal with them? How could you arrange things better? | Could you provide us with an example? |
|  | What is required to achieve this? |  |  |
| Role of the GP | Describe the GP’s role | Please describe the role that your GP plays for you? |  |
| Role of other professions | What role? | -Please tell us what health professionals are involved in providing your health care? | -What people working outside the health system (Self-help groups etc.) provide you with support?  -What kind of support do they provide you with? |
|  | Why do you require such support? |  |  |
| Summary/Conclusion | Summary | -Is there anything else you would like to tell us that is related to this topic?  -Thank you for the interview. |  |
|  | Conclusion |  |  |
