# Supplementary material for: How do middle-aged patients and their healthcare providers manage multimorbidity? Results of a qualitative study
Source: PLoS One. 2023 Aug 31;18(8):e0291065. doi: 10.1371/journal.pone.0291065 (PMC10470875; doi:10.1371/journal.pone.0291065)
Supplement: S3 File — (DOCX) [file pone.0291065.s003.DOCX]

| **ID interview partner** |  |
| --- | --- |
| **Interviewer** |  |
| **Date and time** |  |
| **Interview duration** |  |

**Introduction to interview**

– prior to interview: appointment has been made and a consent form has been signed *–*

- Greet the interview partner, introduce yourself, thank for participation
- **Introduce project:**

*Good morning/afternoon Dr. XXX,*

*Thank you for participating and supporting our research project. My name is XXX and I am a researcher at the Institute of General Practice at Goethe University. The interview is being conducted as part of our MuMiA study, the aim of which is to describe the health care of persons with several chronic diseases from the point of view of patients and healthcare providers. The project is being financed by a charitable foundation entitled* Stiftung Gesundheitswissen *(health knowledge). Other institutions such as health insurers are not involved. Everything you say will be treated confidentially and anonymously. Our discussion will last approximsately 30-40 minutes. We are interested in what you think, which is why we consider you to be an expert. There are no wrong answers. If you don’t object, I would like to record our discussion.*

- Start the recording once you have the interviewee’s consent
- Participation is voluntary
- Data protection – no personal details will be published
- Point out that at the end of the interview, the interviewee will again be asked if all comments may be taken into consideration in the study
- Are there any questions about the procedure?

**The interview**

| **Topic** | **Sub-topic** | **Questions** | **Further questions/reminders** |
| --- | --- | --- | --- |
| First group of topics: **Getting started and description of the subject matter** |  | What patients do you think of when you think of middle-aged patients with several diseases? | Could you describe a typical middle-aged patient with several diseases? |
| Second group of topics: **Characteristics of this group of patients** |  | What is particular about providing healthcare to middle-aged patients with multimorbidity (MM) in comparison to older patients? | What special challenges do you see in providing care to middle-aged patients with MM?  What do you think is especially challenging for middle-aged patients? |
| Third group of topics: **Role as healthcare provider** | From the point of view of the healthcare provider | What role do feel that you play when providing healthcare to this group of patients? |  |
|  |  | How can you support or relieve the burden patients with multimorbidity face during their leisure time, family lives and at work? | How do the diseases influence patients‘ everyday lives? |
|  | From the patient’s point of view | What do patients expect of you as a healthcare provider? | What questions/problems do patients approach you with? |
| Fourth group of topics: **Cooperation/Working relationship** | Interdisciplinary | Who do you involve when providing health care to these patients? | To what extent do you exchange information and agree on an approach with other healthcare providers? |
|  | Social environment | To what extent is the patient’s social network involved? |  |
| Fifth group of topics: **Improving the patients’ health care** | Assessment of patient’s healthcare | What is your opinion about the health care provided to this group of patients? |  |
|  | Comments on the structure of healthcare and the way it is used | How can the healthcare of this group of patients be improved? | What would it take to improve patient safety in the future?  What aspects could be further developed?  What (structural) changes are necessary to improve healthcare for this group of patients?  In your opinion, what possible relationship- and behavior-related preventive measures to avoid negative consequences might make sense? |
|  |  | Do any other helpful sources of information/resources exist that you use when providing care to patients with multimorbidity? | What information is that? Where or from whom do you get the information?  What information would you wish for? |
| Summary/Conclusions | Summary | Is there anything else you would like to tell us that is related to this topic?  May we make further use of what you have told us?  Thank you for the interview. |  |
|  | Conclusion |  |  |
